# A Modular Vaccine Platform Against SARS‐CoV‐2 Based on Self‐Assembled Protein Nanoparticles

**DOI:** 10.1002/advs.202513431

**Published:** 2026-01-14

**Authors:** Seojung Lee, Yejin Jang, Yujin Kim, Yumi Shin, Ji‐Joon Song, Meehyein Kim, Sangyong Jon

**Affiliations:** ^1^ Department of Biological Sciences KAIST Institute for the BioCentury Korea Advanced Institute of Science and Technology (KAIST) Daejeon Republic of Korea; ^2^ Center for Precision Bio‐Nanomedicine Korea Advanced Institute of Science and Technology (KAIST) Daejeon Republic of Korea; ^3^ Infectious Diseases Therapeutic Research Center Korea Research Institute of Chemical Technology (KRICT) Daejeon Republic of Korea

**Keywords:** brucellar bp26, prophylactic vaccine, protein nanoparticles, sars‐cov‐2, spytag/spycatcher

## Abstract

The COVID‐19 pandemic has underscored the urgent need for deployable vaccine platforms capable of rapidly responding to emerging and re‐emerging variants of human coronaviruses. BP26, an outer membrane protein of the zoonotic bacterium *Brucella*, self‐assembles into a highly ordered, barrel‐like nanoparticle that can serve as a vaccine platform, demonstrating immunogenicity against the influenza virus and cancer when combined with specific antigens. Here, we expanded its versatility by incorporating the SpyTag/SpyCatcher pair, enabling modular and site‐specific antigen conjugation via simple mixing. SpyCatcher was genetically fused to BP26, and SpyTag to the receptor binding domain (RBD) of the severe acute respiratory syndrome coronavirus 2 (SARS‐CoV‐2) spike protein, generating RBD‐displaying BP26 nanoparticles (BP26‐RBD). Immunization of mice with BP26‐RBD elicited strong RBD‐specific and neutralizing antibody responses and conferred protection against lethal SARS‐CoV‐2 challenge. This plug‐and‐play nanoparticle design supports antigen production in diverse expression systems, retains antigenic structure, and allows multivalent display, providing a cost‐effective and rapidly adaptable strategy for next‐generation vaccines targeting evolving viral threats.

## Graphical abstract

A modular BP26 nanoparticle vaccine platform incorporating the SpyTag/SpyCatcher system enables high‐density, repetitive antigen display with post‐expression flexibility. Conjugation of the SARS‐CoV‐2 RBD to BP26 nanoparticles elicits strong humoral immunity and confers protection against viral challenge in vivo. This plug‐and‐play system allows for rapid adaptation to emerging variants through facile antigen exchange, multivalent presentation, and compatibility with diverse expression systems.

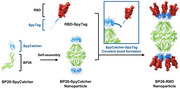

## 1 Introduction

RNA viruses, such as severe acute respiratory syndrome coronavirus 2 (SARS‐CoV‐2), are inherently prone to replication errors, resulting in frequent mutations and the continual emergence of new variants [1, 2, 3, 4, 5, 6]. Some of these, designated as variants of concern, exhibit increased transmissibility and partial resistance to pre‐existing immunity, enabling widespread transmission even among vaccinated or previously infected individuals [7, 8, 9]. These challenges highlight the limitations of conventional vaccine strategies and underscore the urgent need for adaptable platforms that can accommodate antigenic drift while eliciting robust and broadly protective immune responses [10, 11].

Among various approaches, subunit vaccines based on recombinant protein antigens have garnered considerable interest due to their favorable safety profiles, scalability, and amenability to antigenic redesign [12, 13, 14, 15]. In particular, protein nanoparticle‐based vaccine platforms enable the precise genetic loading and high‐density display of antigens, significantly enhancing immunogenicity through multivalent presentation [16, 17, 18]. Virus‐like particles (VLPs), which mimic the structural organization of native viruses and present antigens in a repetitive and ordered fashion, are known to efficiently cross‐link B cell receptors and promote strong humoral immune responses [19, 20, 21]. We recently developed a self‐assembling protein nanoparticle platform derived from BP26, an outer membrane protein of the zoonotic bacterium *Brucella* [22, 23]. This system forms barrel‐like nanoparticles through the assembly of 16 BP26 monomers and leads to dense antigen display via genetic fusion at the C‐terminus [24]. When presenting conserved antigenic peptides from the influenza virus matrix protein 2 (M2), BP26 nanoparticles elicited potent humoral immune responses and conferred cross‐protective immunity against two distinct influenza A/H1N1 isolates in a murine model [22]. Additionally, BP26 fused with tumor neoantigens effectively induced cytotoxic T cell responses and suppressed tumor growth [23], demonstrating its versatile applicability in immunotherapy against both infectious diseases and cancer.

**FIGURE 1:**
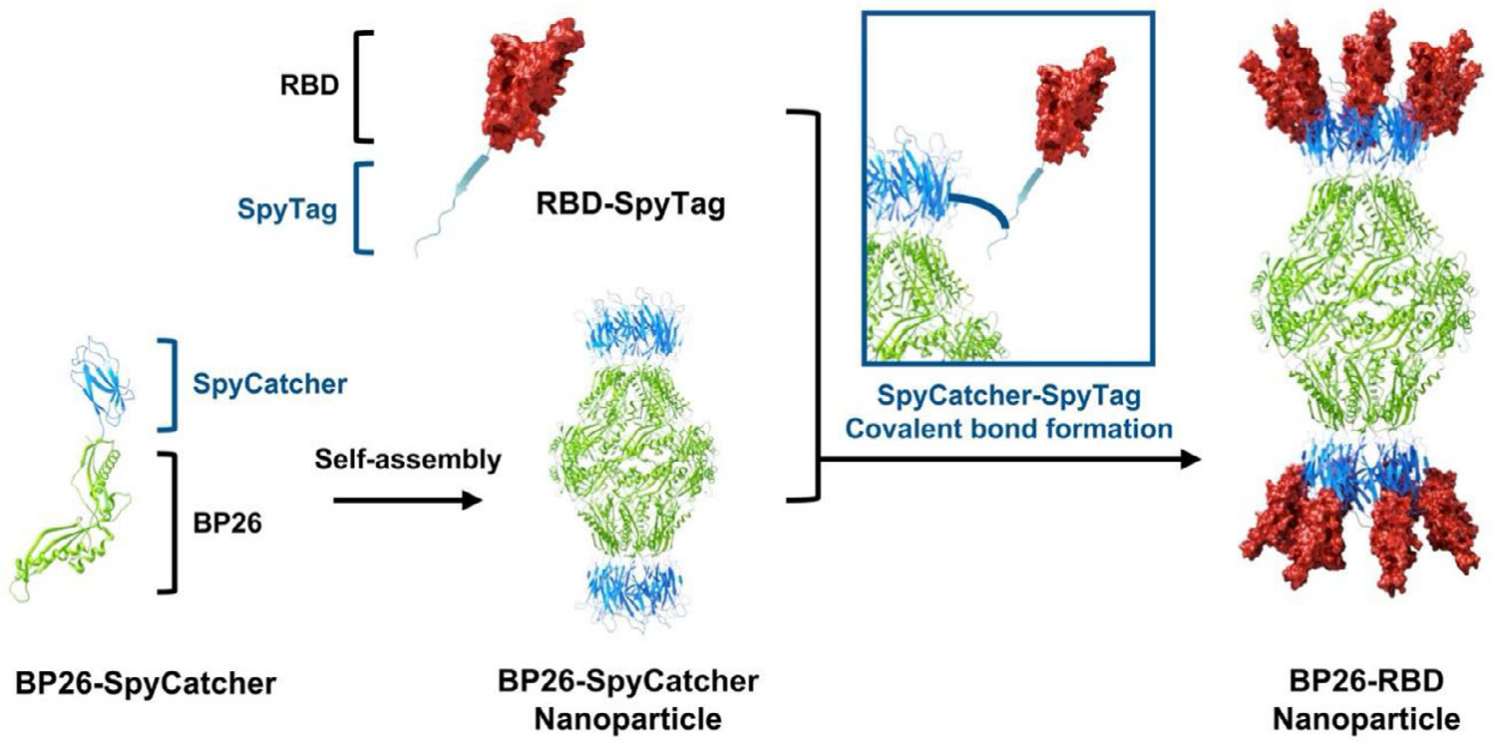
Schematic illustration of receptor binding domain (RBD)‐displaying BP26 nanoparticle (BP26‐RBD) assembly via the SpyTag/SpyCatcher system. BP26 is genetically fused at its C‐terminus with SpyCatcher, and it self‐assembles into a barrel‐shaped nanoparticle scaffold. The RBD of severe acute respiratory syndrome coronavirus 2 (SARS‐CoV‐2), fused with SpyTag, is covalently conjugated to BP26‐SpyCatcher through spontaneous isopeptide bond formation upon simple mixing. This modular strategy enables stable and oriented antigen display on the nanoparticle surface.

To expand the utility of BP26 nanoparticles for targeting rapidly evolving pathogens such as human coronaviruses, we aimed to engineer BP26 as a modular platform that supports facile antigen exchange. Notably, our previous studies confirmed that BP26 nanoparticles maintain structural stability even when fused with antigens of similar size to the BP26 monomer, supporting their potential as a robust scaffold for antigen presentation [22, 23]. To leverage this stability while enabling the attachment of large, independently folded protein subunit antigens, we adopted a covalent conjugation strategy based on the SpyTag/SpyCatcher system. This system facilitates rapid, site‐specific, and irreversible conjugation via spontaneous isopeptide bond formation upon incubation [25, 26]. As a result, it permits efficient post‐expression assembly between nanoparticles and antigens without compromising structural integrity [27, 28, 29, 30, 31]. In this proof‐of‐concept study, we engineered BP26‐SpyCatcher nanoparticles and conjugated them with the receptor‐binding domain (RBD) of SARS‐CoV‐2 S fused to SpyTag (RBD‐SpyTag), creating a modular vaccine candidate (BP26‐RBD) through ratio‐optimized mixing (Figure 1). Immunization of mice with BP26‐RBD elicited potent RBD‐specific neutralizing antibody responses and conferred protection against lethal viral challenge. These findings demonstrate that the BP26‐SpyCatcher platform serves as a robust and versatile antigen delivery system capable of inducing protective humoral immunity, and is well‐suited for mutivalent or rapid‐response vaccine development targeting newly emerging and co‐circulating infectious diseases.

## 2 Results and Discussion

### 2.1 Construction and Characterization of RBD‐Displaying BP26 Nanoparticles (BP26‐RBD)

**FIGURE 2:**
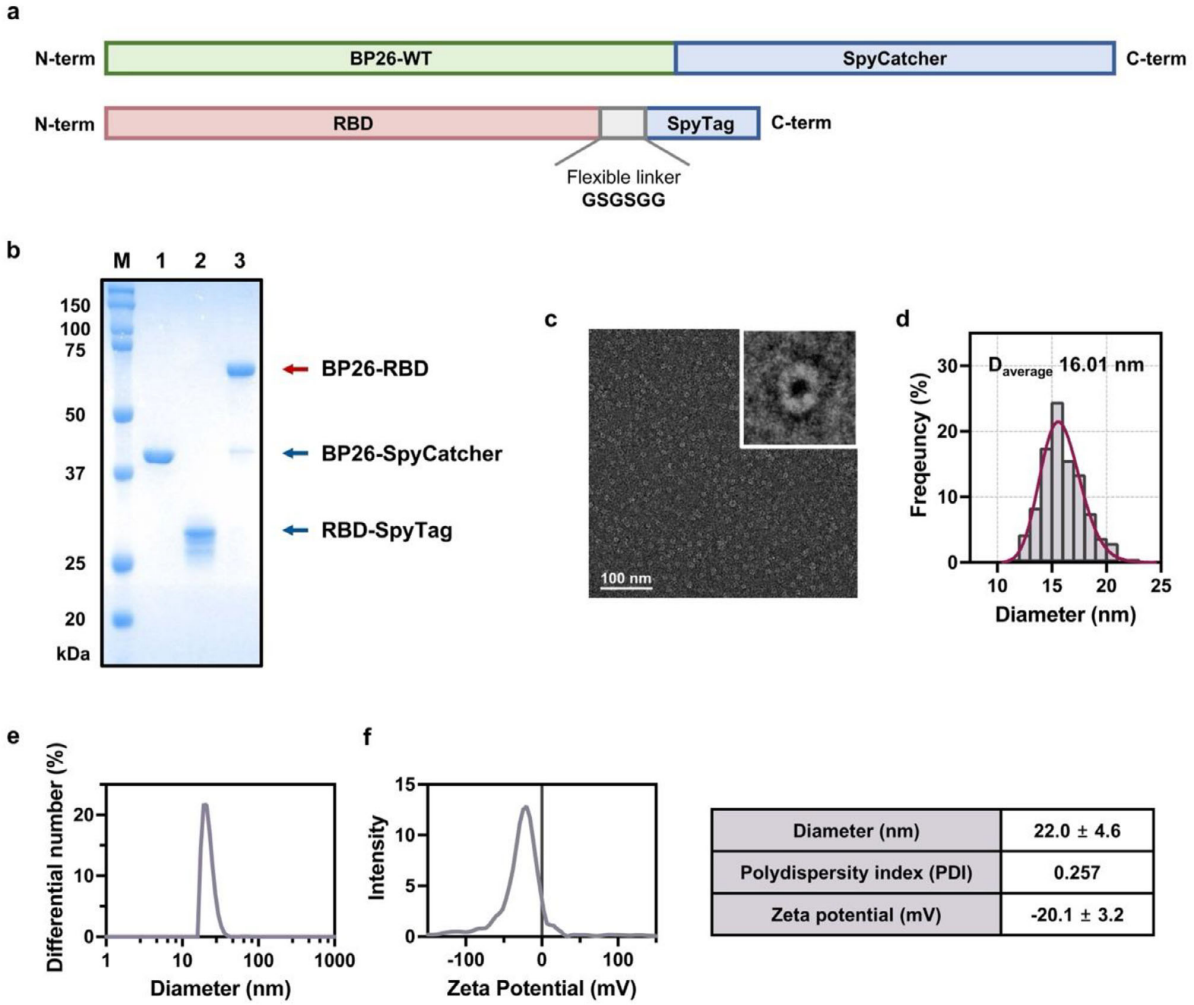
Construction and physicochemical characterization of BP26‐RBD. (a) Constructs of BP26‐SpyCatcher and RBD‐SpyTag. (b) Sodium dodecyl sulfate‐polyacrylamide gel electrophoresis (SDS‐PAGE) analysis of protein markers (lane M), BP26‐SpyCatcher (lane 1), RBD‐SpyTag (lane 2), and assembled BP26‐RBD (lane 3). (c) Representative transmission electron microscopy (TEM) image of BP26‐RBD and (d) corresponding size distribution based on TEM analysis (*n* = 371). Scale bar: 100 nm; magnification: × 43 000. (e) Hydrodynamic diameter and (f) zeta potential of BP26‐RBD measured by dynamic light scattering (DLS). Data are presented as mean ± S.D. (*n* = 3).

To adapt the SpyTag/SpyCatcher system to our BP26 nanoparticle‐based vaccine platform, we designed a modular configuration in which SpyCatcher was genetically fused to BP26 (designated BP26‐SpyCatcher), while SpyTag was appended to the C‐terminus of the RBD of the SARS‐CoV‐2 S protein (Wuhan‐like wild‐type virus) via a flexible GSGSGG linker, generating the RBD‐SpyTag construct (Figure 2a; Table S1). BP26‐SpyCatcher was expressed in *Escherichia coli* (*E. coli*), while RBD‐SpyTag was produced in *Spodoptera frugiperda* (Sf9) cells. Successful expression and purification of both proteins were confirmed by SDS‐PAGE (Figure 2b, lanes 1 and 2). To optimize conjugation conditions, BP26‐SpyCatcher and RBD‐SpyTag were mixed at various molar ratios. The most efficient BP26‐RBD formation was observed at a 4:9 molar ratio, yielding a conjugation efficiency of 89.8% as determined by SDS‐PAGE (Figure S1). Unreacted proteins were removed by size‐exclusion chromatography (SEC), and BP26‐RBD were collected as a distinct peak corresponding to the size of the assembled complex (Figure 2b, lane 3; Figure S2).

Negative‐stain transmission electron microscopy (TEM) revealed that BP26‐RBD formed uniform, hollow, barrel‐shaped nanoparticles with an average diameter of ∼16 nm (Figure 2c,d), closely resembling the morphology of the parent BP26 nanoparticles [24]. Dynamic light scattering (DLS) analysis showed a hydrodynamic diameter of ∼22 nm with a polydispersity index (PDI) of 0.257 (Figure 2e). This increase in hydrodynamic size, compared to previously reported BP26‐based nanoparticles, is consistent with the added molecular weight of the conjugated RBD, reflecting the expected correlation between molecular mass and particle size (Table S2). The zeta potential of BP26‐RBD was approximately −20.1 mV (Figure 2f). Together, these results confirm the successful construction and physicochemical characterization of BP26‐RBD as a homogenous vaccine candidate.

Furthermore, BP26‐RBD demonstrated robust storage stability at 4°C. The hydrodynamic size and polydispersity index remained stable over 4 weeks, as assessed by DLS (Figure S3a). TEM analysis after 4 weeks confirmed that BP26‐RBD preserved its nanoparticle morphology (Figure S3b). SDS‐PAGE showed a single, intact band across all time points, comparable to the −80°C control, indicating no detectable protein degradation (Figure S3c). These collectively confirm that BP26‐RBD maintains structural integrity and particle stability under refrigerated conditions.

### 2.2 Antigen‐Specific Humoral Immune Responses Induced by BP26‐RBD Immunization

**FIGURE 3:**
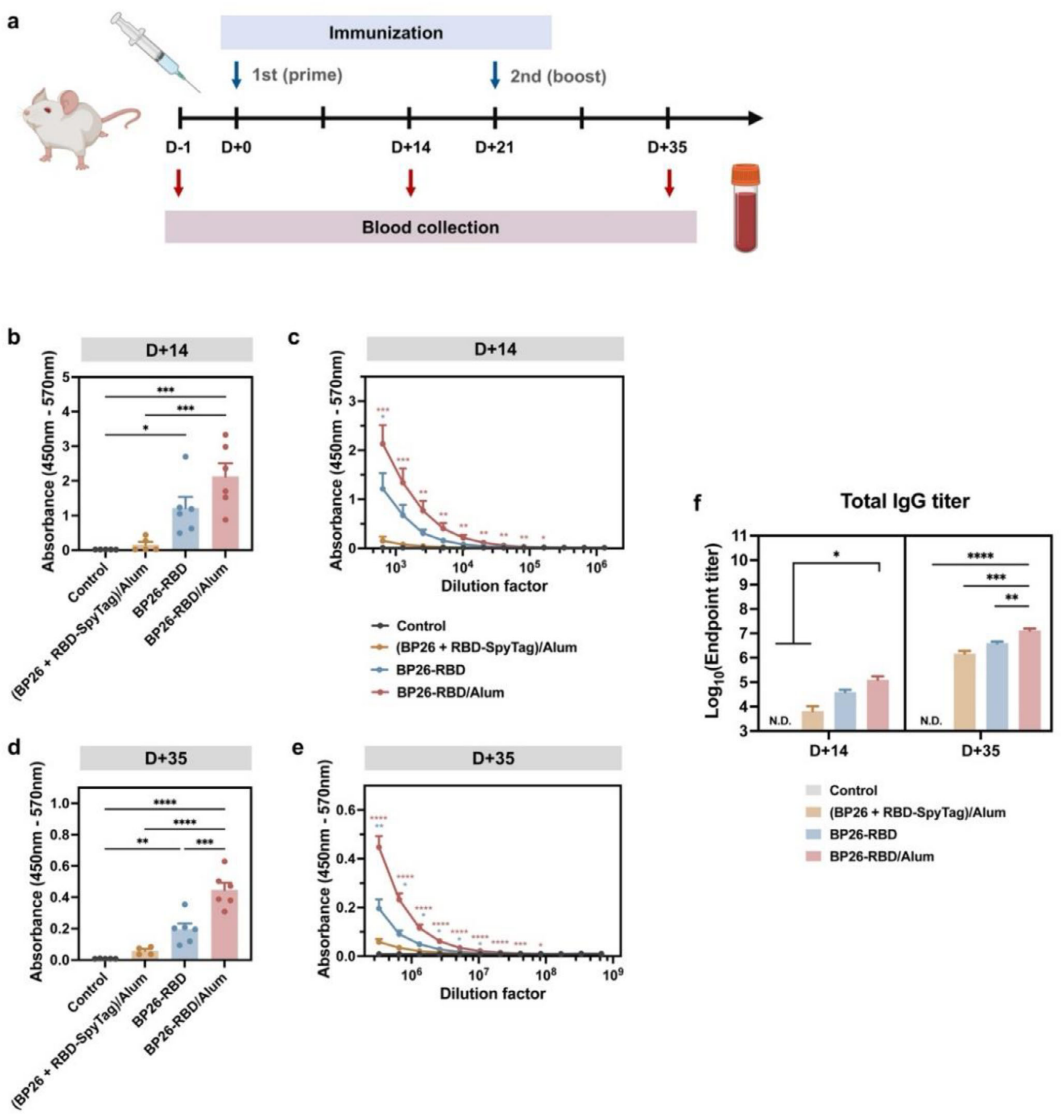
Antigen‐specific humoral immune responses induced by BP26‐RBD immunization. (a) Immunization and blood collection schedule. BALB/c mice (*n* = 5–6 per group) were immunized subcutaneously twice at a 3‐week interval with each vaccine formulation containing an equivalent RBD dose (10 µg per injection). Blood samples were collected retro‐orbitally 1 day prior to the prime immunization and two weeks after both the prime and boost doses. RBD‐specific immunoglobulin G (IgG) levels were quantified by enzyme‐linked immunosorbent assay (ELISA). (b) Serum RBD‐specific IgG titers at a 1:640 dilution measured on day 14 post‐prime. (c) IgG titers determined by serial two‐fold dilution of day 14 sera. (d) Serum RBD‐specific IgG titers at a 1:327,680 dilution on day 35 post‐boost. (e) Serial dilution curves of day 35 sera. (f) Endpoint titers of RBD‐specific total IgG following the prime and boost immunizations. The endpoint titer was defined as the highest dilution yielding an absorbance value at least twofold higher than the pre‐immune baseline. Data are presented as mean ± S.E.M. Statistical significance was determined by one‐way ANOVA followed by Tukey's post hoc test (^*^ *p* < 0.05, ^**^ *p* < 0.01, ^***^ *p* < 0.001, ^****^ *p* < 0.0001). (Illustration created with BioRender.com.).

To evaluate the immunogenicity of BP26‐RBD, BALB/c mice were immunized subcutaneously following a prime‐boost regimen with a 3‐week interval (Figure 3a). Sera were collected 1 day before the initial immunization and 2 weeks after each dose to assess antigen‐specific antibody responses. Mice receiving a simple mixture of BP26 nanoparticles and RBD‐SpyTag, co‐administered with alum adjuvant, produced negligible levels of anti‐RBD IgG antibodies after both the prime and boost immunizations (Figure 3b–e). In contrast, immunization with BP26‐RBD led to significantly elevated RBD‐specific antibody titers, particularly in the presence of alum adjuvant, indicating a robust humoral immune response. Serum antibody levels were quantified by endpoint titer analysis, defined as the highest serum dilution yielding a signal at least two‐fold above the pre‐immune baseline (Figure 3c,e; Figure S4a,c). Following the booster dose, endpoint titers increased by approximately 100‐200‐fold relative to the prime dose (Figure 3f; Figure S4b,d). Notably, BP26‐RBD vaccination with alum resulted in ∼19‐fold and 9‐fold increases in endpoint titers after the prime and boost, respectively, compared to the unassembled mixture of BP26 and RBD‐SpyTag. These findings demonstrate that covalent antigen display via the SpyTag/SpyCatcher system enables BP26 nanoparticles to elicit strong antigen‐specific antibody responses, establishing this platform as a potent inducer of humoral immunity. To assess the modularity of the BP26 platform, an alternative antigen, the fibronectin extra domain B (EDB), was further conjugated to BP26 nanoparticles, employing the SpyTag/SpyCatcher system (Figure S5a–d). Vaccination with EDB‐displaying BP26 nanoparticles (BP26‐EDB) resulted in robust antigen‐specific antibody responses, supporting the broad applicability of the BP26 nanoparticles as a versatile vaccine platform (Figure S5e–g).

### 2.3 Virus‐Neutralizing Activity of Serum Antibodies From Immunized Mice

**FIGURE 4:**
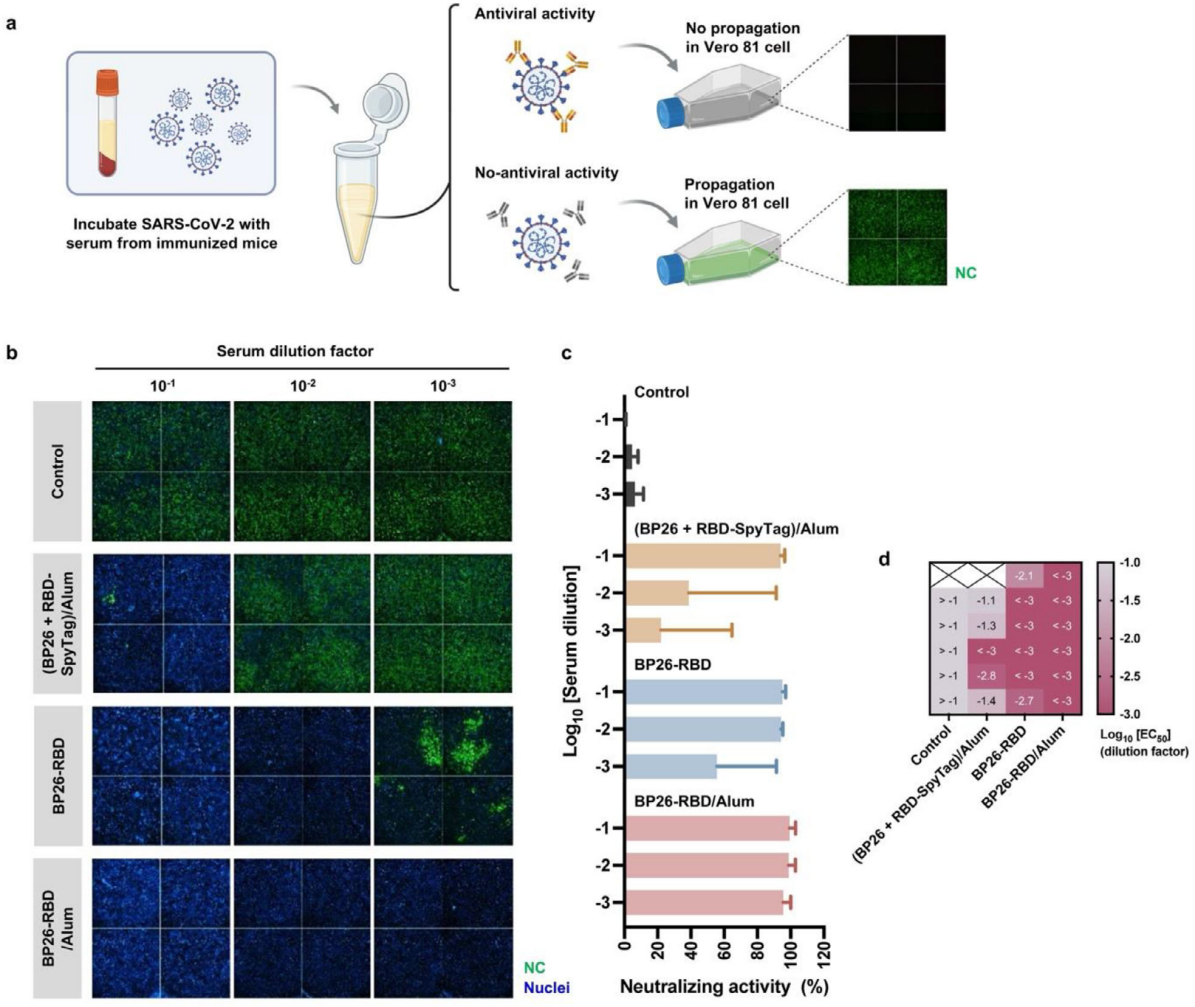
Virus‐neutralizing activity of serum antibodies induced by BP26‐RBD immunization. (a) Schematic overview of the experimental workflow for assessing SARS‐CoV‐2 neutralization. Sera collected two weeks after the booster immunization (*n* = 5–6 mice per group) were mixed at a 1:1 volume ratio with 300 infectious SARS‐CoV‐2 particles and incubated prior to infection of Vero cells. Neutralizing activity was assessed by immunofluorescence detection of nucleocapsid (NC) protein expression using a primary anti‐NC antibody and Alexa Fluor 488‐labeled secondary antibody. Nuclei were counterstained with DAPI. (Illustration created with BioRender.com.) (b) Representative fluorescence images of infected Vero cells treated with serum‐virus mixtures (technical duplicates). NC protein (green) indicates viral infection; nuclei are shown in blue (DAPI). (c) Quantification of virus neutralization using serial dilutions of post‐boost serum. (d) Half maximal effective concentration (EC_50_) values of individual serum samples calculated from the dilution curves. Data are presented as mean ± S.D.

Although protein subunit or peptide vaccines elicit antigen‐specific antibodies, their neutralizing capacity is often limited by spatially suboptimal epitope display or by antibody targeting non‐neutralizing domains [32, 33, 34]. To assess the functional capacity of antibodies induced by BP26‐RBD vaccination, we evaluated serum neutralizing activity against a Wuhan‐like SARS‐CoV‐2 strain, which served as the source of the RBD in our vaccine construct. Sera were collected 2 weeks after the booster immunization, as outlined in Figure 3a. Serial dilutions of immune sera (ranging from 1:10–1:1000 in 10‐fold increments) were pre‐incubated with live virus and then applied to the infection of Vero 81 cells. In principle, the presence of neutralizing antibodies blocks viral propagation, whereas in their absence, infection proceeds and results in the expression of nucleocapsid (NC) protein, which can be immunofluorescence (Figure 4a). To ensure assay fidelity, Sotrovimab, a clinically approved neutralizing antibody, was included as a positive control (Figures S6e and S7e) [35]. Compared to sera from mice immunized with the unassembled mixture of BP26 and RBD‐SpyTag with alum, sera from non‐adjuvanted BP26‐RBD‐immunized mice showed a remarkable reduction in NC expression, even at higher dilution factors (1/100 and 1/1,000), indicating the induction of potent neutralizing antibodies (Figure 4b; Figure S6a–c). Specifically, in the group immunized with the unassembled mixture ((BP26 + RBD‐SpyTag)/Alum), neutralizing activity was undetectable at the 1:100 dilution in three out of five mice (Figure S6b). In contrast, all mice immunized with BP26‐RBD exhibited clear neutralization at this dilution, regardless of alum adjuvant use (Figure S6c). Notably, sera from the BP26‐RBD/Alum group retained robust neutralizing activity even at the highest tested dilution (1:1000) (Figure S6d). Neutralization efficacy was quantified by measuring the NC‐associated fluorescent intensity, normalized to DAPI‐stained nuclei, enabling accurate comparison across groups (Figure 4c; Figure S7). The 50% effective concentration (EC_50_) values, calculated and derived from serum dilution curves and presented as a color‐code matrix, revealed that all BP26‐RBD/Alum‐vaccinated mice achieved EC_50_ values below the 1:1000 dilution. Collectively, these results demonstrate that covalent antigen display on BP26 nanoparticles via SpyTag/SpyCatcher conjugation effectively induces potent neutralizing antibodies. Moreover, our findings corroborate that the engineered vaccine construct is compatible with the conventional adjuvant, alum, underscoring its immunological advantage over the unconjugated vaccine formulation represented by (BP26 + RBD‐SpyTag)/Alum.

### 2.4 Protective Efficacy Against SARS‐CoV‐2 Challenge

**FIGURE 5:**
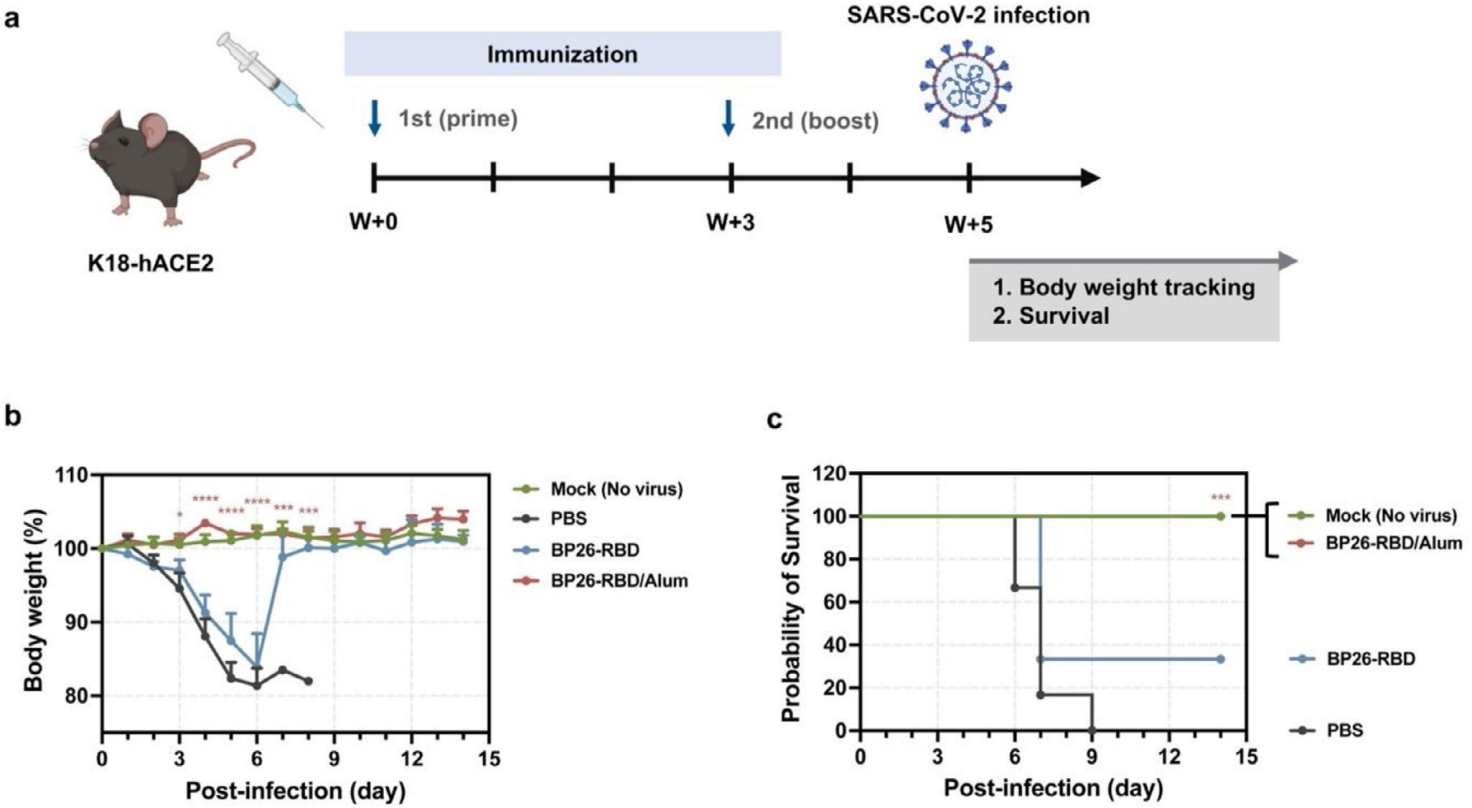
Protective efficacy of BP26‐RBD vaccination against SARS‐CoV‐2 challenge. (a) Schematic of the immunization and viral challenge protocol in K18‐hACE2 transgenic mice. Mice (*n* = 6 per group) were immunized subcutaneously twice at a 3‐week interval with each vaccine formulation. Two weeks after the booster dose, mice were intranasally challenged with SARS‐CoV‐2 at 30 × median lethal dose 50% (MLD_50_). (Illustration created with BioRender.com.) (b) Changes in body weight and (c) survival rates were monitored over time following viral challenge to evaluate vaccine‐mediated protection. Data are presented as mean ± S.E.M. Statistical significance was determined relative to the PBS group (^*^ *p* < 0.05, ^**^ *p* < 0.01, ^***^ *p* < 0.001, ^****^ *p* < 0.0001; multiple unpaired two‐tailed t‐tests with Holm‐Sidak correction). Survival curves were analyzed using the Log‐rank (Mantel‐Cox) test.

To evaluate the in vivo protective efficacy of BP26‐RBD vaccination, we conducted a viral challenge study using K18‐hACE2 transgenic mice, which express human ACE2 under the human keratin 18 (K18) promoter and are susceptible to SARS‐CoV‐2 infection [36, 37]. Based on earlier findings demonstrating robust induction of neutralizing antibodies (Figure 4), we designed a challenge experiment to explore the prophylactic efficacy of unadjuvanted BP26‐RBD and its alum‐adjuvanted formulation. K18‐hACE2 mice were immunized subcutaneously with BP26‐RBD or BP26‐RBD/Alum following a prime‐boost regimen at a 3‐week interval (Figure 5a). Two weeks after the boost dose, mice were challenged intranasally with an extremely lethal dose (30 × 50% mouse lethal dose (MLD_50_)). Body weight and survival were monitored daily for 14 days post‐infection to assess vaccine‐mediated protection. Unimmunized mice began to exhibit progressive weight loss by day 2 post‐infection, resulting in 100% mortality by day 9 (Figure 5b,c; Figure S8). In contrast, BP26‐RBD‐immunized mice showed partial protection, with two out of six mice (33.3%) surviving. Remarkably, co‐administration of BP26‐RBD with alum provided complete protection, with all mice surviving and maintaining stable body weight, comparable to the unchallenged mock control group. To assess dose responsiveness, mice were immunized with BP26‐RBD/Alum at three RBD antigen doses (5, 10, and 20 µg) and challenged with SARS‐CoV‐2. All dose groups showed complete protection, with full survival and no detectable weight loss (Figure S9), stressing that even the lowest tested dose conferred reliable protection in this lethal challenge model. These findings underscore the strong protective efficacy of the alum‐adjuvanted BP26‐RBD and demonstrate the translational potential of the BP26‐SpyCatcher platform for SARS‐CoV‐2, as well as its scalability to other emerging coronavirus variants through multivalent or universal vaccine strategies.

## 3 Conclusion

We developed a modular nanoparticle‐based vaccine strategy leveraging the self‐assembling BP26 scaffold and SpyTag/SpyCatcher covalent conjugation, and evaluated its effectiveness against SARS‐CoV‐2 through a BP26‐RBD construct as a proof of concept. Immunization with BP26‐RBD elicited potent RBD‐specific neutralizing antibody responses and conferred considerable protection in a lethal viral challenge model. Importantly, co‐administration with alum markedly enhanced efficacy, supporting compatibility with clinically approved adjuvants. A major strength of this design lies in its architectural flexibility. The BP26‐SpyCatcher component is efficiently produced in *E. coli* and stockpiled as a universal carrier, while antigenic targets, expressed in eukaryotic systems to ensure proper folding and post‐translational modifications [38, 39, 40, 41, 42], can be site‐specifically conjugated via SpyTag for stable and multivalent display. This configuration facilitates streamlined development of cost‐effective subunit vaccines with preserved antigenic structure and strong immunogenicity [43, 44, 45]. Additionally, it could allow for presentation of multiple antigens, which may diversify the humoral repertoire and improve cross‐reactive efficacy. Incorporation of variant‐specific or pan‐coronavirus antigens into BP26 nanoparticle vaccines may offer broader coverage against antigenically distinct strains [46]. In light of the ongoing evolution of SARS‐CoV‐2 and the emergence of novel zoonotic viruses such as HKU5‐CoV lineage 2 merbecovirus [47], the BP26‐SpyCatcher system serves as a flexible vaccine framework. Its plug‐and‐play assembly, structural robustness, and antigenic adaptability make it a promising candidate for next‐generation immunization options targeting rapidly mutating RNA viruses and supporting global pandemic preparedness efforts. Additional investigations, such as assessing how BP26 enhances B cell responses through antigen‐mediated B cell receptor clustering, or how it is internalized and processed by antigen‐presenting cells to initiate downstream T cell activation, could offer deeper insight into how this scaffold interfaces with the immune system. In particular, clarifying how its barrel‐like architecture, with directional and repetitive antigen presentation, promotes immune activation may help illuminate why BP26, paired with a modular conjugation system, is an efficient and adaptable antigen‐delivery platform.

## 4 Experimental Section

### 4.1 Animals, Cells, and Viruses

Female BALB/c mice (5 weeks old) were obtained from Orient Bio (Seongnam, Korea), and female K18‐hACE2 transgenic mice (B6.Cg‐Tg(K18‐ACE2)2Prlmn/J; 6 weeks old) were purchased from The Jackson Laboratory (Bar Harbor, ME, USA). Mice were housed under specific pathogen‐free (SPF) conditions at a controlled temperature (23°C) and relative humidity (45%–53%). Animals were maintained in groups of 5–6 per cage in individually ventilated cages (IVCs) with soft bedding, and were provided ad libitum access to standard rodent chow and water. Following a 1‐week acclimation period, mice weighing 16–18 g were randomly assigned to experimental groups. All procedures involving animals were performed under isoflurane anesthesia to minimize pain and distress. Experimental protocols were approved by the Animal Care and Use Committees of the Korea Advanced Institute of Science and Technology (KAIST) (Approval Nos. KA2021‐009 and KA2025‐061‐v1) and the Institutional Animal Care and Use Committee (IACUC) of the Korea Research Institute of Chemical Technology (KRICT) (Approval No. 2024–6D‐05‐01).

African green monkey kidney epithelial cells (Vero; ATCC CCL‐81) were obtained from the American Type Culture Collection (ATCC; Manassas, VA, USA). Cells were cultured in Dulbecco's Modified Eagle Medium (DMEM; HyClone, South Logan, UT, USA) supplemented with 10% fetal bovine serum (FBS; Atlas Biologicals, Fort Collins, CO, USA) and maintained at 37°C in a humidified incubator with 5% CO_2_.

The wild‐type SARS‐CoV‐2 strain (hCoV‐19/Korea/KCDC06/2020) was kindly provided by the National Culture Collection for Pathogens (NCCP), Republic of Korea. All experiments involving infectious SARS‐CoV‐2 were conducted in biosafety level 3 (BSL‐3) facilities at KRICT.

### 4.2 Cloning, Expression, and Purification of BP26‐SpyCatcher and RBD‐SpyTag Recombinant Proteins

DNA sequences encoding BP26‐SpyCatcher and RBD‐SpyTag were synthesized and cloned into expression vectors by GenScript (Piscataway, NJ, USA). For BP26‐SpyCatcher expression, the SpyCatcher sequence was genetically fused to the C‐terminus of the BP26. The gene was codon‐optimized for expression in *Escherichia coli* (*E. coli*) and cloned into the pET‐28a(+)‐TEV vector using NdeI and XhoI restriction sites at the 5’ and 3’ ends, respectively. The resulting plasmid was transformed into *E. coli* BL21 CodonPlus (DE3)‐RILP cells. Transformants were cultured in LB broth supplemented with kanamycin and chloramphenicol. Recombinant protein expression was induced with 1 mm isopropyl 𝛽‐D‐1‐thiogalactopyranoside (IPTG) at 18°C for 16–18 h. Recombinant proteins were initially purified using Ni‐NTA affinity chromatography (Qiagen, Hilden, Germany), followed by size‐exclusion chromatography using a Superdex 200 26/60 column (Cytiva, Marlborough, MA, USA). The expression and purity of the recombinant BP26‐SpyCatcher protein were confirmed by SDS‐PAGE with Coomassie Brilliant Blue staining.

For expression of RBD‐SpyTag protein, the SpyTag sequence was fused to the C‐terminus of the RBD derived from the Wuhan‐like SARS‐CoV‐2 strain, with a GSGSGG flexible linker between the two domains. To enable secretion in insect cells, a gp67 signal peptide was added at the N‐terminus. A TEV protease cleavage site (ENLYFQG) was inserted to allow removal of the gp67 signal peptide and His‐tag during purification. The gene was codon‐optimized for expression in *Spodoptera frugiperda* (Sf9) cells and cloned into the pFastBac1 vector using BamHI and XhoI restriction sites. The recombinant plasmid was transformed into *E. coli* DH10Bac for bacmid generation. The resulting bacmid encoding the gp67‐RBD‐SpyTag was transfected into Sf9 cells to generate recombinant baculovirus, which was subsequently amplified through serial infection of Sf9 cells. Cells were harvested 42 h post‐infection and lysed, and the recombinant RBD‐SpyTag protein was purified using Ni‐NTA affinity chromatography (Qiagen), followed by purification with Superdex 75 15/60 size‐exclusion chromatography (Cytiva). The gp67 signal peptide was cleaved before size‐exclusion chromatography. Expression and purification of the RBD‐SpyTag protein were confirmed by SDS‐PAGE with Coomassie Brilliant Blue staining.

### 4.3 Construction of BP26‐RBD Proteins

BP26‐SpyCatcher and RBD‐SpyTag were mixed to form the BP26‐RBD complex. To determine the optimal complexation ratio, BP26‐SpyCatcher (fixed at 16 pmol) was mixed with increasing molar amounts of RBD‐SpyTag (9, 18, 36, 54, and 72 pmol, corresponding to 1‐, 2‐, 4‐, 6‐, and 8‐fold molar excesses relative to 9 pmol). SDS‐PAGE analysis identified the 4:9 molar ratio (BP26‐SpyCatcher:RBD‐SpyTag) as optimal, and all subsequent BP26‐RBD complexes were prepared using this ratio. Conjugation efficiency was quantified by measuring the band intensities of BP26‐SpyCatcher before and after conjugation using the Fiji distribution of Image J (version 1.53) and calculated using the equation: Conjugation efficiency (%) = 100 × {1 – (Band intensity of BP26‐SpyCatcher after conjugation)/(Band intensity of BP26‐SpyCatcher before conjugation)}. To remove unbound proteins and isolate the BP26‐RBD complex, the reaction mixture was subjected to Superdex 6 10/300 size‐exclusion chromatography (Cytiva). The final BP26‐RBD complex was validated by SDS‐PAGE to confirm its formation and purity. Endotoxins were removed from the protein solution using High‐Capacity Endotoxin Removal Spin Columns (Thermo Fisher Scientific, Waltham, MA, USA), and the endotoxin levels were quantified using a chromogenic Limulus Amebocyte Lysate (LAL) endotoxin assay kit (GenScript).

### 4.4 Characterization of BP26‐RBD

BP26‐RBD were negatively stained with 1% uranyl acetate and examined by TEM using a Philips TECNAI F20 instrument (Philips Electronic Instrument Corp., Mahwah, NJ, USA). The hydrodynamic size, polydispersity index (PDI), and zeta potential were measured by DLS using a Zeta‐potential & Particle Size Analyzer (Otsuka Electronics, Osaka, Japan) according to the manufacturer's instructions.

### 4.5 Immunization for Antibody Titration

Six‐week‐old female BALB/c mice were immunized using a prime‐boost regimen with 3‐week intervals between doses. Mice were randomly assigned to four groups and immunized via subcutaneous injection into both footpads with the following vaccine formulations: (1) PBS vehicle control, (2) a simple mixture of BP26 and RBD‐SpyTag with alum adjuvant, (3) BP26‐RBD, and (4) BP26‐RBD with alum adjuvant. Each formulation was prepared to deliver 9.4 µg BP26, 10.8 µg RBD‐SpyTag, or 25.1 µg BP26‐RBD per mouse. Particularly, for groups receiving RBD‐containing formulations, the antigen dose was adjusted to ensure the administration of 10 µg of RBD per injection. For groups administered with alum adjuvant, the commercially available aluminum hydroxide adjuvant Rehydragel HP (Reheis Chemicals, Berkeley Heights, NJ, USA), was used. Antigen solutions were mixed with a 30% aluminum hydroxide suspension at a 1:1 volume ratio and incubated at room temperature (RT) for 1 h to allow formulation.

### 4.6 Quantification of RBD‐Specific Antibody Production

RBD‐specific antibody responses were assessed in mice immunized as described above. Blood samples were collected via retro‐orbital bleeding at three time points: day ‐1 (1 day before the first immunization), day 14 (2 weeks after the prime), and day 35 (2 weeks after the boost). Sera were isolated using serum‐separating tubes (BD, NJ, USA) by centrifugation at 2000 relative centrifugal force (rcf) for 10 min. RBD‐specific antibody titers were determined by enzyme‐linked immunosorbent assay (ELISA). High‐binding 96‐well plates (Corning, NY, USA) were coated with recombinant human coronavirus SARS‐CoV‐2 Spike glycoprotein RBD protein (Abcam, Cambridge, UK) at a concentration of 1 µg/mL and incubated overnight at 4°C. The plates were washed three times with phosphate‐buffered saline containing 0.05% Tween 20 (PBST), and then blocked with PBST containing 5% bovine serum albumin (BSA) for 1 h at RT. After washing, serially diluted serum samples (prepared in PBS with 5% BSA) were added and incubated for 2 h at RT. Plates were washed three times with PBST and incubated with horseradish peroxidase (HRP)‐conjugated goat anti‐mouse IgG (H+L) antibody (1:10 000 dilution; Thermo Fisher Scientific; catalog no. 31430) for 2 h at RT. Following additional washing and complete removal of residual liquid, tetramethylbenzidine (TMB) substrate solution (R&D Systems, MN, USA) was added and incubated for 20 min at RT to allow color development. The reaction was terminated by the addition of 2N sulfuric acid, and absorbance was measured at 450 nm using a microplate reader. One outlier from the group treated with a simple mixture of BP26 and RBD‐SpyTag with alum adjuvant was excluded based on Grubb's test (α = 0.05).

### 4.7 In Vitro Neutralizing Activity Assay

Sera collected from immunized mice, as described above, were used to evaluate neutralizing antibody levels. Vero cells were seeded in 96‐well plates at a density of 3 × 10^4^ cells per well and incubated overnight. SARS‐CoV‐2 (at a multiplicity of infection (MOI) of 0.01) was pre‐incubated with serially diluted serum samples (1:10, 1:100, and 1:1000) for 1 h at 37°C. The mixtures of virus and sera were then added to the Vero cells. At 24 h post‐infection, cells were fixed and permeabilized with a chilled acetone‐methanol solution (1:3 ratio) for 10 min at RT. Viral nucleocapsid (NC) protein was detected using a rabbit anti‐NC antibody (Genetex, Irvine, CA, USA; catalog no. GTX135357), followed by incubation with an Alexa Fluor 488‐conjugated goat anti‐rabbit IgG secondary antibody (Invitrogen, Waltham, MA, USA; catalog no. A‐11008). Cell nuclei were counterstained with 4’,6‐diamidino‐2‐phenylindole (DAPI; Invitrogen). Neutralizing efficacy was quantified by measuring the reduction in NC‐derived fluorescence signal, normalized to DAPI intensity. The 50% effective concentration (EC_50_) was defined as the serum dilution that resulted in a 50% reduction in SARS‐CoV‐2 infection relative to the untreated control.

### 4.8 Protective Efficacy Against SARS‐CoV‐2 Challenge

Six‐week‐old female K18‐hACE2 mice were immunized following the prime‐boost regimen with a 3‐week interval, as described above. Briefly, mice were randomly divided into four groups and immunized via subcutaneous injection into both footpads with the following vaccine formulations: (1) PBS vehicle for two groups, one serving as a mock control and the other as an infection control, (2) BP26‐RBD containing 10 µg of RBD per mouse, and (3) BP26‐RBD with alum adjuvant with increasing doses of RBD at 5, 10, or 20 µg per mouse. Two weeks after the final immunization, mice were intranasally challenged with SARS‐CoV‐2 (2 × 10^4^ plaque‐forming units in 50 µL PBS per mouse, corresponding to 30 × MLD_50_ [48]. Body weight and mortality were monitored daily for 14 days post‐infection. Mice that exhibited ≥ 25% loss in body weight were humanely euthanized and included in the mortality count.

### 4.9 Statistical Analysis

In vivo data are presented as the mean ± standard error of the mean (S.E.M.) and other data as the mean ± standard deviation (S.D.). TEM size distributions were fitted to a lognormal model using nonlinear regression, and geometric mean and S.D. were obtained. Group comparisons involving more than two conditions were analyzed by one‐way analysis of variance (ANOVA) with Tukey's multiple comparisons. Pairwise comparisons were performed using multiple unpaired two‐tailed t‐tests with Holm‐Sidak correction. Survival curves were analyzed using the Log‐rank (Mantel‐Cox) test. Data distribution was assessed using the Shapiro‐Wilk normality test to justify the use of parametric tests. In all analyses, 95% confidence intervals (95% CI) were calculated, and *P*‐values less than 0.05 were considered statistically significant. All statistical analyses were conducted using GraphPad Prism 10 (GraphPad Software, CA, USA).

## Author Contributions

S.L. and Y.J. contributed equally to this work. The manuscript was written through the contribution of all authors. S.L., Y.J., Y.K., J.S., M.K., and S.J. conceived the project and designed the experiments. S.L. and Y.J. mainly performed the in vitro and in vivo experiments. Y.S. contributed to the preparation of the recombinant proteins. S.L., Y.J., J.S., M.K., and S.J. analyzed the data. S.L. and S.J. wrote the manuscript, and J.S. and M.K. revised the draft.

## Funding

This work was supported by the National Research Foundation of Korea (NRF) grant funded by the Korea government (MSIT) (RS‐2018‐NR030951 and RS‐2022‐NR067905 to S. Jon), and by an intramural grant from the Korea Research Institute of Chemical Technology (KRICT; KK2532‐30 to M. Kim).

## Research Resource

The SARS‐CoV‐2 strain (hCoV‐19/Korea/KCDC06/2020; NCCP 43328) was provided by the National Culture Collection for Pathogens (NCCP), Republic of Korea.

## Conflicts of Interest

The authors declare no conflicts of interest.

## Supporting information




**Supporting File**: advs73765‐sup‐0001‐SuppMat.docx.

## Data Availability

The data that support the findings of this study are available from the corresponding authors upon reasonable request.

## References

[1] J. W. Drake and J. J. Holland , “Mutation Rates Among RNA Viruses,” Proceedings of the National Academy of Sciences 96 (1999): 13910–13913. 10.1073/pnas.96.24.13910 PMC24164 10570172

[2] A. S. Lauring , J. Frydman , and R. Andino , “The Role of Mutational Robustness in RNA Virus Evolution,” Nature Reviews Microbiology 11 (2013): 327–336. 23524517 10.1038/nrmicro3003 PMC3981611

[3] E. Domingo and J. Holland , “RNA Virus Mutations and Fitness for Survival,” Annual Review of Microbiology 51 (1997): 151–178. 10.1146/annurev.micro.51.1.151 9343347

[4] M. M. Lamers and B. L. Haagmans , “SARS‐CoV‐2 Pathogenesis,” Nature Reviews Microbiology 20 (2022): 270–284. 35354968 10.1038/s41579-022-00713-0

[5] F. Krammer , G. J. Smith , R. Fouchier , et al., “Influenza,” Nature Reviews Disease Primers 4 (2018): 3. 10.1038/s41572-018-0002-y PMC7097467 29955068

[6] R. Uraki , B. Korber , M. S. Diamond , and Y. Kawaoka , “SARS‐CoV‐2 Variants: Biology, Pathogenicity, Immunity and Control,” Nature Reviews Microbiology 4 (2025): 8–28. 10.1038/s41579-025-01255-x PMC12973737 41214236

[7] A. M. Carabelli , T. P. Peacock , L. G. Thorne , et al., “SARS‐CoV‐2 Variant Biology: Immune Escape, Transmission and Fitness,” Nature Reviews Microbiology (2023), 21, 162–177. 36653446 10.1038/s41579-022-00841-7 PMC9847462

[8] V. N. Petrova and C. A. Russell , “The Evolution of Seasonal Influenza Viruses,” Nature Reviews Microbiology 16 (2018): 47–60. 29109554 10.1038/nrmicro.2017.146

[9] W. F. Garcia‐Beltran , E. C. Lam , K. S. Denis , et al., “Multiple SARS‐CoV‐2 Variants Escape Neutralization by Vaccine‐Induced Humoral Immunity,” Cell 184 (2021): 2372–2383. 33743213 10.1016/j.cell.2021.03.013 PMC7953441

[10] A. Gupta , A. Rudra , K. Reed , R. Langer , and D. G. Anderson , “Advanced Technologies for the Development of Infectious Disease Vaccines,” Nature Reviews Drug Discovery 23 (2024): 914–938. 39433939 10.1038/s41573-024-01041-z PMC13310450

[11] F. Zhao , X. Zai , Z. Zhang , J. Xu , and W. Chen , “Challenges and Developments in Universal Vaccine Design Against SARS‐CoV‐2 Variants,” NPJ Vaccines 7 (2022): 167. 36535982 10.1038/s41541-022-00597-4 PMC9761649

[12] V. P. Chavda , E. N. H. K. Ghali , P. C. Balar , et al., “Protein Subunit Vaccines: Promising Frontiers Against COVID‐19,” Journal of Controlled Release 366 (2024): 761–782. 38219913 10.1016/j.jconrel.2024.01.017

[13] M. Heidary , V. H. Kaviar , M. Shirani , et al., “A Comprehensive Review of the Protein Subunit Vaccines Against COVID‐19,” Frontiers in Microbiology 13 (2022): 927306. 35910658 10.3389/fmicb.2022.927306 PMC9329957

[14] Y. Zhang , J. Gao , W. Xu , et al., “Advances in Protein Subunit Vaccines Against H1N1/09 Influenza,” Frontiers in Immunology 15 (2024): 1499754. 39650643 10.3389/fimmu.2024.1499754 PMC11621219

[15] G. Wang , A. K. Verma , J. Shi , et al., “Universal Subunit Vaccine Protects Against Multiple SARS‐CoV‐2 Variants and SARS‐CoV,” NPJ Vaccines 9 (2024): 133. 39054338 10.1038/s41541-024-00922-z PMC11272943

[16] B. Nguyen and N. H. Tolia , “Protein‐Based Antigen Presentation Platforms for Nanoparticle Vaccines,” NPJ Vaccines 6 (2021): 70. 33986287 10.1038/s41541-021-00330-7 PMC8119681

[17] N. Butkovich , E. Li , A. Ramirez , A. M. Burkhardt , and S. W. Wang , “Advancements in Protein Nanoparticle Vaccine Platforms to Combat Infectious Disease,” Nanomedicine and Nanobiotechnology 13 (2021): 1681. 10.1002/wnan.1681 PMC8052270 33164326

[18] H. Wu , R. Weng , J. Li , et al., “Self‐Assembling Protein Nanoparticle Platform for Multivalent Antigen Delivery in Vaccine Development,” International Journal of Pharmaceutics 676 (2025): 125597. 40233885 10.1016/j.ijpharm.2025.125597

[19] M. O. Mohsen and M. F. Bachmann , “Virus‐Like Particle Vaccinology, From Bench to Bedside,” Cellular & Molecular Immunology 19 (2022): 993–1011. 35962190 10.1038/s41423-022-00897-8 PMC9371956

[20] S. Nooraei , H. Bahrulolum , Z. S. Hoseini , et al., “Virus‐Like Particles: Preparation, Immunogenicity and Their Roles as Nanovaccines and Drug Nanocarriers,” Journal of Nanobiotechnology 19 (2021): 59. 33632278 10.1186/s12951-021-00806-7 PMC7905985

[21] X. Hao , F. Yuan , and X. Yao , “Advances in Virus‐Like Particle‐based SARS‐CoV‐2 Vaccines,” Frontiers in Cellular and Infection Microbiology 14 (2024): 1406091. 38988812 10.3389/fcimb.2024.1406091 PMC11233461

[22] S. Kang , Y. Kim , Y. Shin , J.‐J. Song , and S. Jon , “Antigen‐Presenting, Self‐Assembled Protein Nanobarrels as an Adjuvant‐Free Vaccine Platform Against Influenza Virus,” ACS Nano 15 (2021): 10722–10732. 34114799 10.1021/acsnano.1c04078

[23] Y. Kim , S. Lee , J. Yoon , et al., “Neoantigen‐Displaying Protein Nanoparticles as a Therapeutic Cancer Vaccine Against Melanoma,” Advanced Healthcare Materials 14 (2025): 2404316. 10.1002/adhm.202404316 39713909

[24] D. Kim , J. Park , S. J. Kim , et al., “Brucella Immunogenic BP26 Forms a Channel‐Like Structure,” Journal of Molecular Biology 425 (2013): 1119–1126. 23353825 10.1016/j.jmb.2013.01.015

[25] B. Zakeri , J. O. Fierer , E. Celik , et al., “Peptide Tag Forming a Rapid Covalent Bond to a Protein, Through Engineering a Bacterial Adhesin,” Proceedings of the National Academy of Sciences 109 (2012): E690–E697. 10.1073/pnas.1115485109 PMC3311370 22366317

[26] A. H. Keeble , P. Turkki , S. Stokes , et al., “Approaching Infinite Affinity Through Engineering of Peptide–Protein Interaction,” Proceedings of the National Academy of Sciences 116 (2019): 26523–26533. 10.1073/pnas.1909653116 PMC6936558 31822621

[27] T. K. Tan , P. Rijal , R. Rahikainen , et al., “A COVID‐19 Vaccine Candidate Using SpyCatcher Multimerization of the SARS‐CoV‐2 Spike Protein Receptor‐Binding Domain Induces Potent Neutralising Antibody Responses,” Nature Communications 12 (2021): 542. 10.1038/s41467-020-20654-7 PMC7822889 33483491

[28] V. Lampinen , S. Gröhn , S. Soppela , V. Blazevic , V. P. Hytönen , and M. M. Hankaniemi , “SpyTag/SpyCatcher Display of Influenza M2e Peptide on norovirus‐Like Particle Provides Stronger Immunization than Direct Genetic Fusion,” Frontiers in Cellular and Infection Microbiology 13 (2023): 1216364. 37424789 10.3389/fcimb.2023.1216364 PMC10323135

[29] W. Wang , Z. Liu , X. Zhou , et al., “Ferritin Nanoparticle‐Based SpyTag/SpyCatcher‐Enabled Click Vaccine for Tumor Immunotherapy,” Nanomedicine: Nanotechnology, Biology and Medicine 16 (2019): 69–78. 30529790 10.1016/j.nano.2018.11.009

[30] Z.‐H. Liu , Z.‐F. Deng , Y. Lu , W.‐H. Fang , and F. He , “A Modular and Self‐adjuvanted Multivalent Vaccine Platform Based on Porcine Circovirus Virus‐Like Nanoparticles,” Journal of Nanobiotechnology 20 (2022): 493. 36424615 10.1186/s12951-022-01710-4 PMC9685936

[31] T. D. Nguyen , H. D. Le , G. C. Dang , et al., “A Combined Adjuvant and Ferritin Nanocage Based Mucosal Vaccine against Streptococcus pneumoniae Induces Protective Immune Responses in a Murine Model,” Nature Communications 16 (2025): 2871. 10.1038/s41467-025-58115-8 PMC11933286 40128220

[32] P. A.‐B. Weidenbacher , E. Waltari , I. de los Rios Kobara , et al., “Converting Non‐neutralizing SARS‐CoV‐2 Antibodies Into Broad‐spectrum Inhibitors,” Nature Chemical Biology 18 (2022): 1270–1276. 36076082 10.1038/s41589-022-01140-1 PMC9596371

[33] T. F. Rogers , F. Zhao , D. Huang , et al., “Isolation of Potent SARS‐CoV‐2 Neutralizing Antibodies and Protection From Disease in a Small Animal Model,” Science 369 (2020): 956–963. 32540903 10.1126/science.abc7520 PMC7299280

[34] L. Liu , P. Wang , M. S. Nair , et al., “Potent Neutralizing Antibodies Against Multiple Epitopes on SARS‐CoV‐2 Spike,” Nature 584 (2020): 450–456. 32698192 10.1038/s41586-020-2571-7

[35] A. Gupta , Y. Gonzalez‐Rojas , E. Juarez , et al., “Early Treatment for Covid‐19 With SARS‐CoV‐2 Neutralizing Antibody Sotrovimab,” New England Journal of Medicine 385 (2021): 1941–1950. 34706189 10.1056/NEJMoa2107934

[36] P. B. McCray Jr , L. Pewe , C. Wohlford‐Lenane , et al., “Lethal Infection of K18‐hACE2 Mice Infected with Severe Acute respiratory Syndrome Coronavirus,” Journal of Virology 81 (2007): 813. 17079315 10.1128/JVI.02012-06 PMC1797474

[37] W. Dong , H. Mead , L. Tian , et al., “The K18‐human ACE2 Transgenic Mouse Model Recapitulates Non‐severe and Severe COVID‐19 in Response to an Infectious Dose of the SARS‐CoV‐2 Virus,” Journal of Virology 96 (2022): 00964. 10.1128/JVI.00964-21 PMC8754221 34668775

[38] G. L. Rosano and E. A. Ceccarelli , “Recombinant Protein Expression in Escherichia coli: Advances and Challenges,” Frontiers in Microbiology 5 (2014): 172. 24860555 10.3389/fmicb.2014.00172 PMC4029002

[39] A. Schütz , F. Bernhard , N. Berrow , et al., “A Concise Guide to Choosing Suitable Gene Expression Systems for Recombinant Protein Production,” STAR Protocols 4 (2023): 102572. 37917580 10.1016/j.xpro.2023.102572 PMC10643540

[40] T. A. Kost , J. P. Condreay , and D. L. Jarvis , “Baculovirus as Versatile Vectors for Protein Expression in Insect and Mammalian Cells,” Nature Biotechnology 23 (2005): 567–575. 10.1038/nbt1095 PMC3610534 15877075

[41] T. U. Gerngross , “Advances in the Production of human Therapeutic Proteins in Yeasts and Filamentous Fungi,” Nature Biotechnology 22 (2004): 1409–1414. 10.1038/nbt1028 15529166

[42] V. Gomord and L. Faye , “Posttranslational Modification of Therapeutic Proteins in Plants,” Current Opinion in Plant Biology 7 (2004): 171–181. 15003218 10.1016/j.pbi.2004.01.015

[43] G. Walsh and R. Jefferis , “Post‐translational Modifications in the Context of Therapeutic Proteins,” Nature Biotechnology 24 (2006): 1241–1252. 10.1038/nbt1252 17033665

[44] R. Ojha and V. K. Prajapati , “Cognizance of Posttranslational Modifications in Vaccines: A Way to Enhanced Immunogenicity,” Journal of Cellular Physiology 236 (2021): 8020–8034. 34170014 10.1002/jcp.30483 PMC8427110

[45] J. Park and J. A. Champion , “Effect of Antigen Structure in Subunit Vaccine Nanoparticles on Humoral Immune Responses,” ACS Biomaterials Science & Engineering 9 (2023): 1296–1306. 36848229 10.1021/acsbiomaterials.2c01516 PMC10015428

[46] Y.‐F. Kang , C. Sun , J. Sun , et al., “Quadrivalent Mosaic HexaPro‐bearing Nanoparticle Vaccine Protects Against Infection of SARS‐CoV‐2 Variants,” Nature Communications 13 (2022): 2674. 10.1038/s41467-022-30222-w PMC9106700 35562337

[47] J. Chen , W. Zhang , Y. Li , et al., “Bat‐Infecting Merbecovirus HKU5‐CoV Lineage 2 Can Use human ACE2 as a Cell Entry Receptor,” Cell 188 (2025): 1729–1742. 39970913 10.1016/j.cell.2025.01.042

[48] M. Kim , M. K. Lee , I. Jo , et al., “Identification of 4′‐Thiouridine as an Orally Available Antiviral Agent Targeting both RdRp and NiRAN Functions of SARS‐CoV‐2 Nsp12,” Journal of Medicinal Chemistry 68 (2025): 12414–12433. 40512093 10.1021/acs.jmedchem.4c02874

