# Supplementary material for: A Modular Vaccine Platform Against SARS‐CoV‐2 Based on Self‐Assembled Protein Nanoparticles
Source: Adv Sci (Weinh). 2026 Jan 14;13(10):e13431. doi: 10.1002/advs.202513431 (PMC12915080; doi:10.1002/advs.202513431)
Supplement: Supplementary file 1 — Supporting File: advs73765‐sup‐0001‐SuppMat.docx. [file ADVS-13-e13431-s001.docx]

Supporting Information

**A Modular Vaccine Platform Against SARS-CoV-2 Based on Self-Assembled Protein Nanoparticles**

*Seojung Lee^†^, Yejin Jang^†^, Yujin Kim, Yumi Shin, Ji-Joon Song*, Meehyein Kim* and Sangyong Jon**

S. Lee, Dr. Y. Kim, Y. Shin, Prof. Dr. J. Song, Prof. Dr. S. Jon

Department of Biological Sciences, KAIST Institute for the BioCentury

Korea Advanced Institute of Science and Technology (KAIST)

291 Daehak-ro, Daejeon 34141, Republic of Korea

E-mail: [songj@kaist.ac.kr](mailto:songj@kaist.ac.kr); [syjon@kaist.ac.kr](mailto:syjon@kaist.ac.kr)

S. Lee, Dr. Y. Kim, Prof. Dr. S. Jon

Center for Precision Bio-Nanomedicine

Korea Advanced Institute of Science and Technology (KAIST)

291 Daehak-ro, Daejeon 34141, Republic of Korea

Dr. Y. Jang, Dr. M. Kim

Infectious Diseases Therapeutic Research Center

Korea Research Institute of Chemical Technology (KRICT)

141 Gajeong-ro, Daejeon 34114, Republic of Korea

E-Mail: [mkim@krict.re.kr](mailto:mkim@krict.re.kr)

^†^ Seojung Lee and Yejin Jang contributed equally to this work.

**Supplementary Figures**


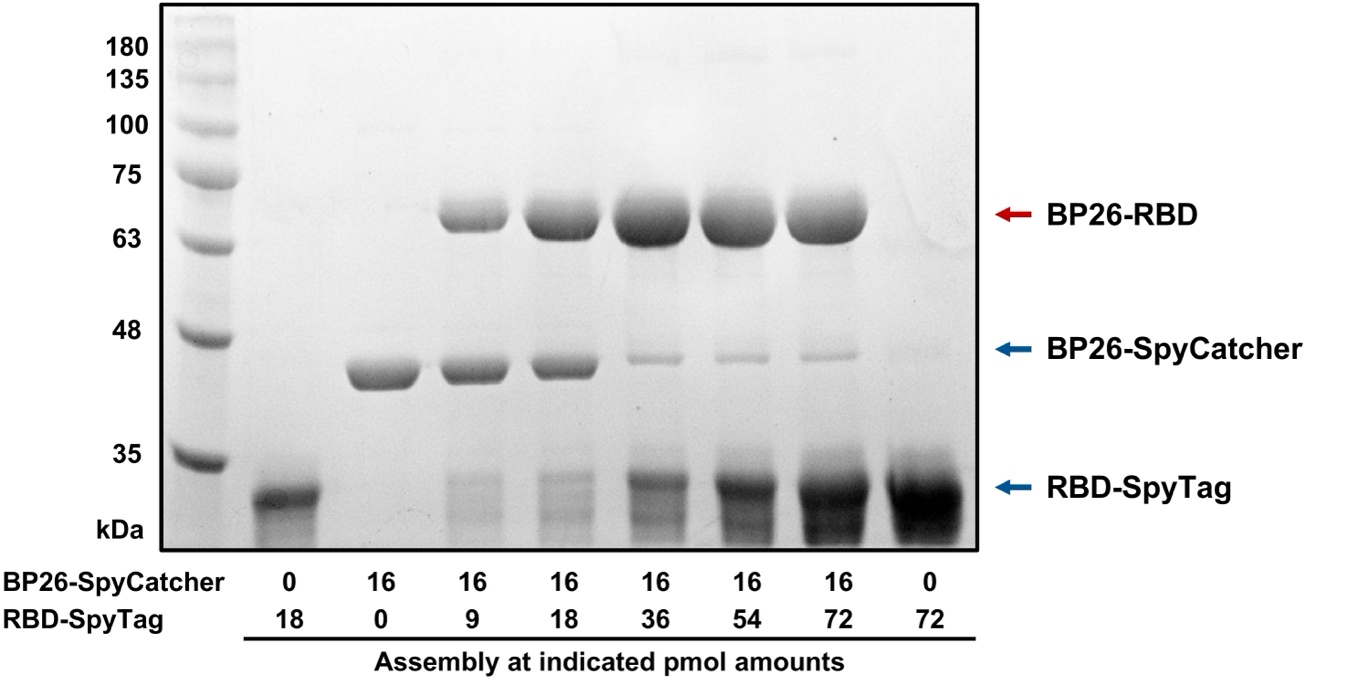


**Figure S1. Optimization of BP26-RBD assembly conditions.** BP26-SpyCatcher (16 pmol) was incubated with increasing molar amounts of RBD-SpyTag (9, 18, 36, 54, and 72 pmol) to determine the optimal conjugation ratio for nanoparticle assembly. The reaction mixtures were analyzed by sodium dodecyl sulfate-polyacrylamide gel electrophoresis (SDS-PAGE) to assess the efficiency of BP26-RBD complex formation. Arrows indicate the positions of unreacted BP26-SpyCatcher, free RBD-SpyTag, and the BP26-RBD conjugate.


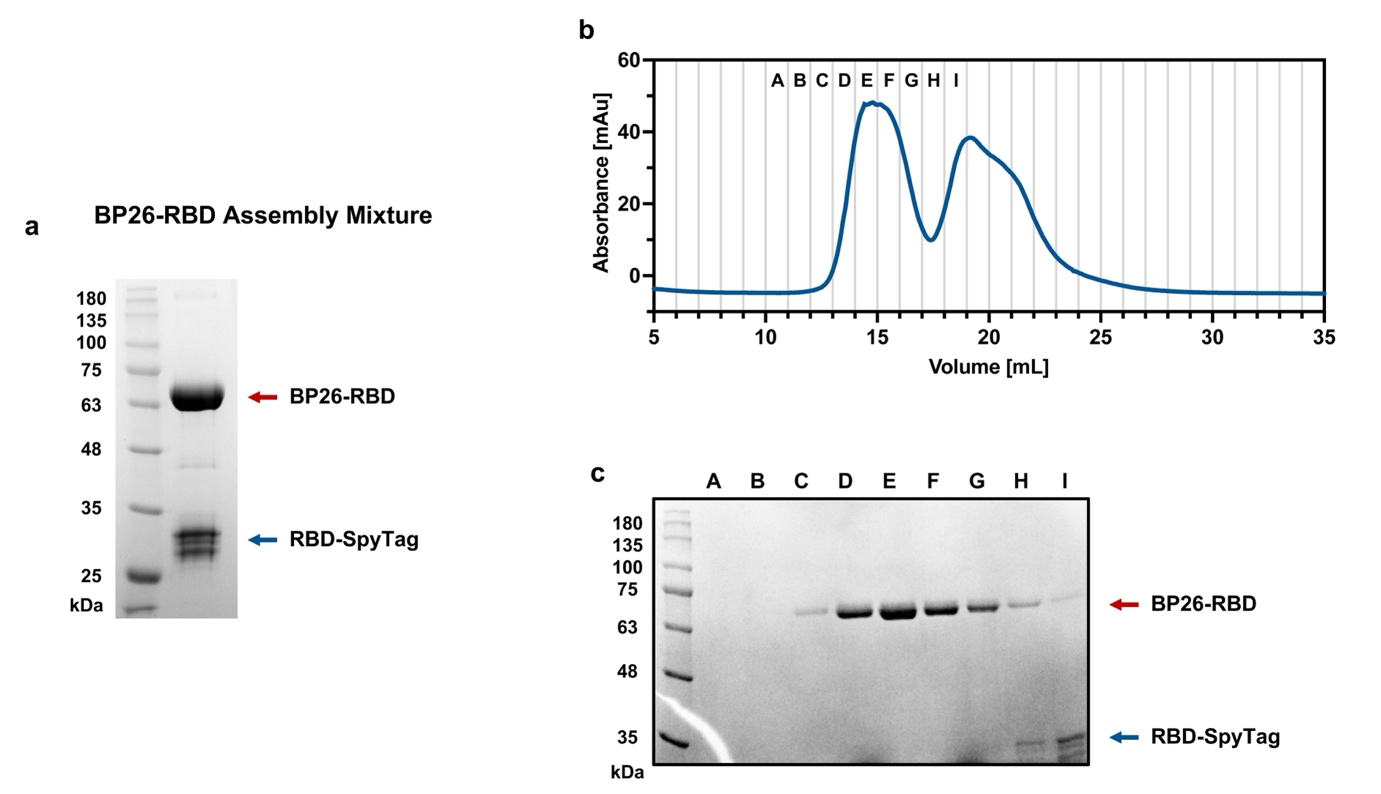


**Figure S2. Purification of BP26-RBD by size-exclusion chromatography (SEC). a)** BP26-SpyCatcher and RBD-SpyTag were mixed at the optimized molar ratio of 4:9, and conjugation efficiency was confirmed by SDS-PAGE. **b)** Unreacted RBD-SpyTag was removed by SEC, and the corresponding elution profile is shown. **c)** SDS-PAGE analysis of individual SEC fractions. Fractions containing BP26-RBD conjugates were identified, pooled, and used for subsequent experiments.


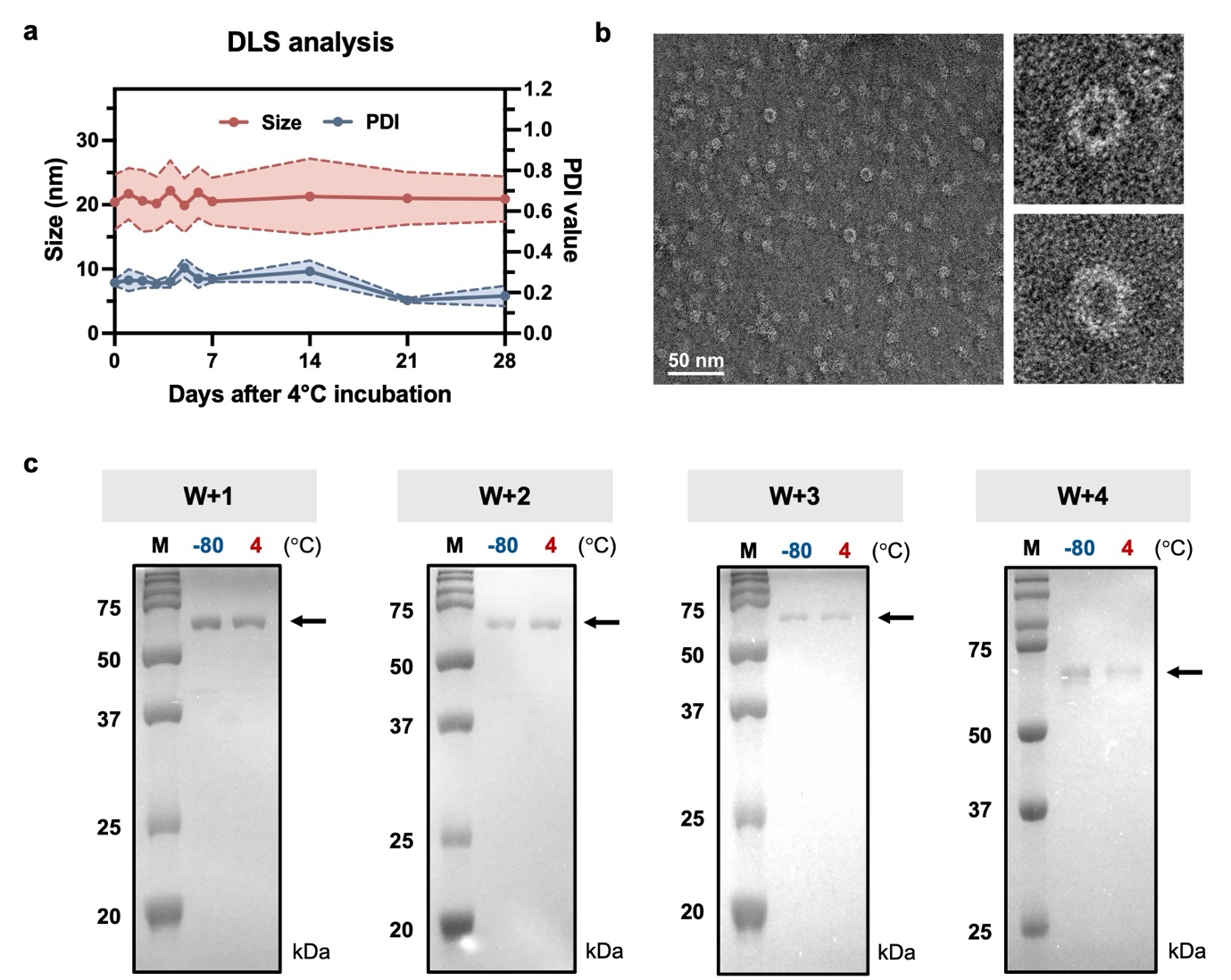


**Figure S3.** Storage stability of BP26-RBD at 4 °C. BP26-RBD were stored in PBS at 4 °C for 4 weeks. a) Hydrodynamic size and polydispersity index of BP26-RBD, measured daily during the first week and weekly thereafter by dynamic light scattering (DLS). Data are presented as means ± S.D. (n = 4). b) Representative transmission electron microscopy (TEM) images of BP26-RBD after 4 weeks of storage at 4 °C. c) Sodium dodecyl sulfate-polyacrylamide gel electrophoresis (SDS-PAGE) analysis of BP26-RBD after 1, 2, 3, and 4 weeks of incubation at 4 °C, compared with the sample stored at -80 °C, showing a single, intact band without detectable degradation.


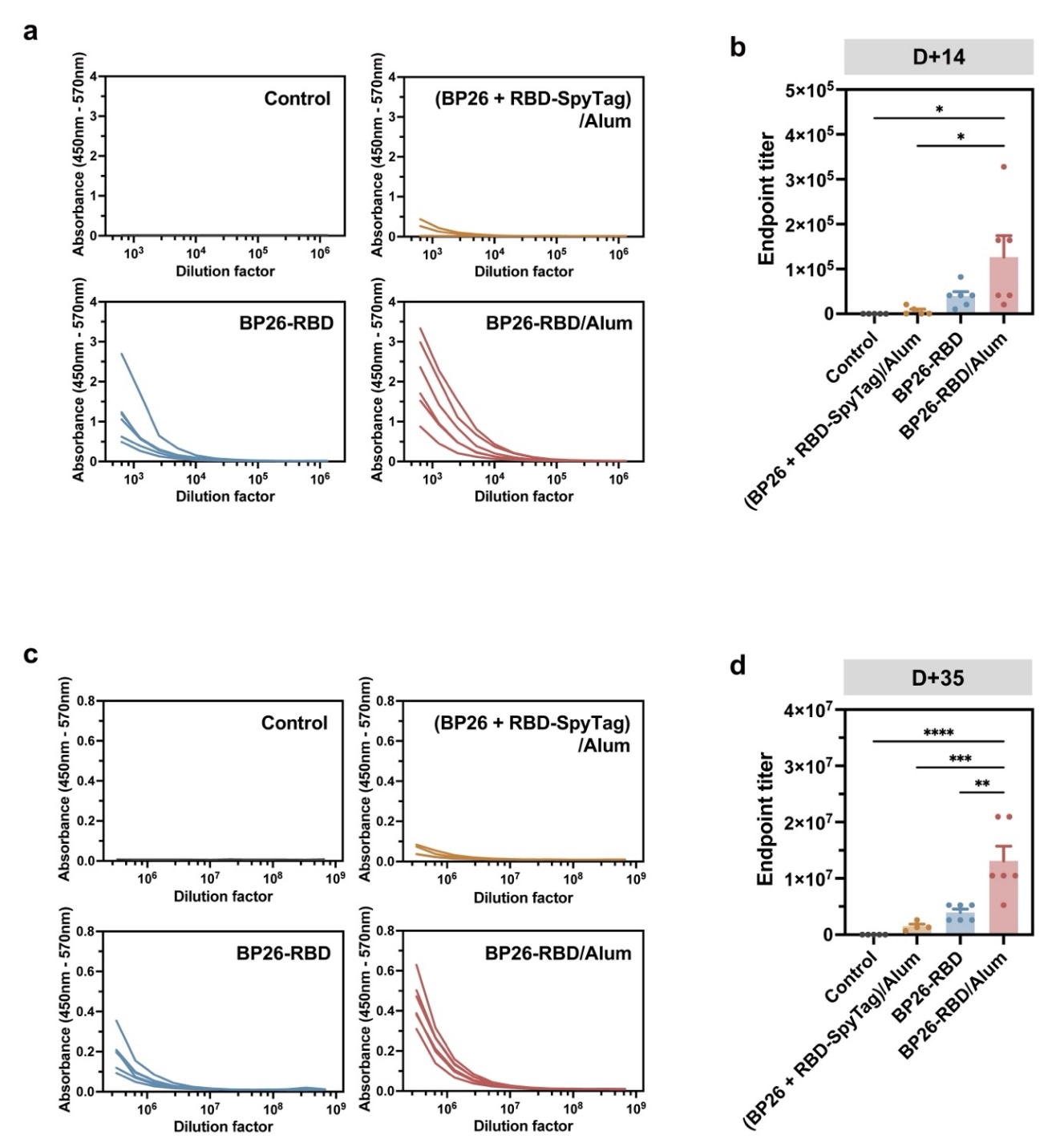


**Figure S4. RBD-specific antibody titers in individual mice following prime and boost immunizations. a)** Individual immunoglobulin G (IgG) titers determined by serial two-fold dilutions of sera collected on day 14 (two weeks post-prime). **b)** Corresponding endpoint titers derived from the dilution curves. **c)** Individual IgG titers from sera collected on day 35 (two weeks post-boost). **d)** Corresponding endpoint titers. The endpoint titer was defined as the highest serum dilution yielding an absorbance at least twofold above the pre-immune background. Data are presented as mean ± S.E.M. Statistical significance was assessed by one-way ANOVA with Tukey’s post hoc test (**P* < 0.05, ***P* < 0.01, ****P* < 0.001, *****P* < 0.0001).

**Figure S5.** Construction and antigen-specific humoral immune responses of EDB-displaying BP26 nanoparticles (BP26-EDB). a) Constructs of BP26-SpyCatcher and EDB-SpyTag. b) Purification of BP26-EDB by size-exclusion chromatography (SEC). c) Sodium dodecyl sulfate-polyacrylamide gel electrophoresis (SDS-PAGE) analysis of individual SEC fractions. Fractions of BP26-EDB conjugates were identified, pooled and used for subsequent experiments. d) Representative transmission electron microscopy (TEM) image of BP26-EDB. e) Immunization and blood collection schedule. BALB/c mice (n = 6 per group) were immunized subcutaneously twice at a 1-week interval with each vaccine formulation containing equivalent EDB dose (10 μg per injection). Blood samples were collected retro-orbitally one day prior to the prime immunization and a week after both the prime and boost doses. EDB-specific immunoglobulin G (IgG) levels were quantified by enzyme-linked immunosorbent assay (ELISA). f) Serum EDB-specific IgG titers at a 1:100 dilutions on week 1 post-prime and g) at a 1:1,000 dilutions on week 2 post boost. Data are presented as mean ± S.E.M. Statistical significance was determined by one-way ANOVA followed by Tukey’s post hoc test (**P* < 0.05, ***P* < 0.01, ****P* < 0.001, *****P* < 0.0001). (Illustration created with BioRender.com.)


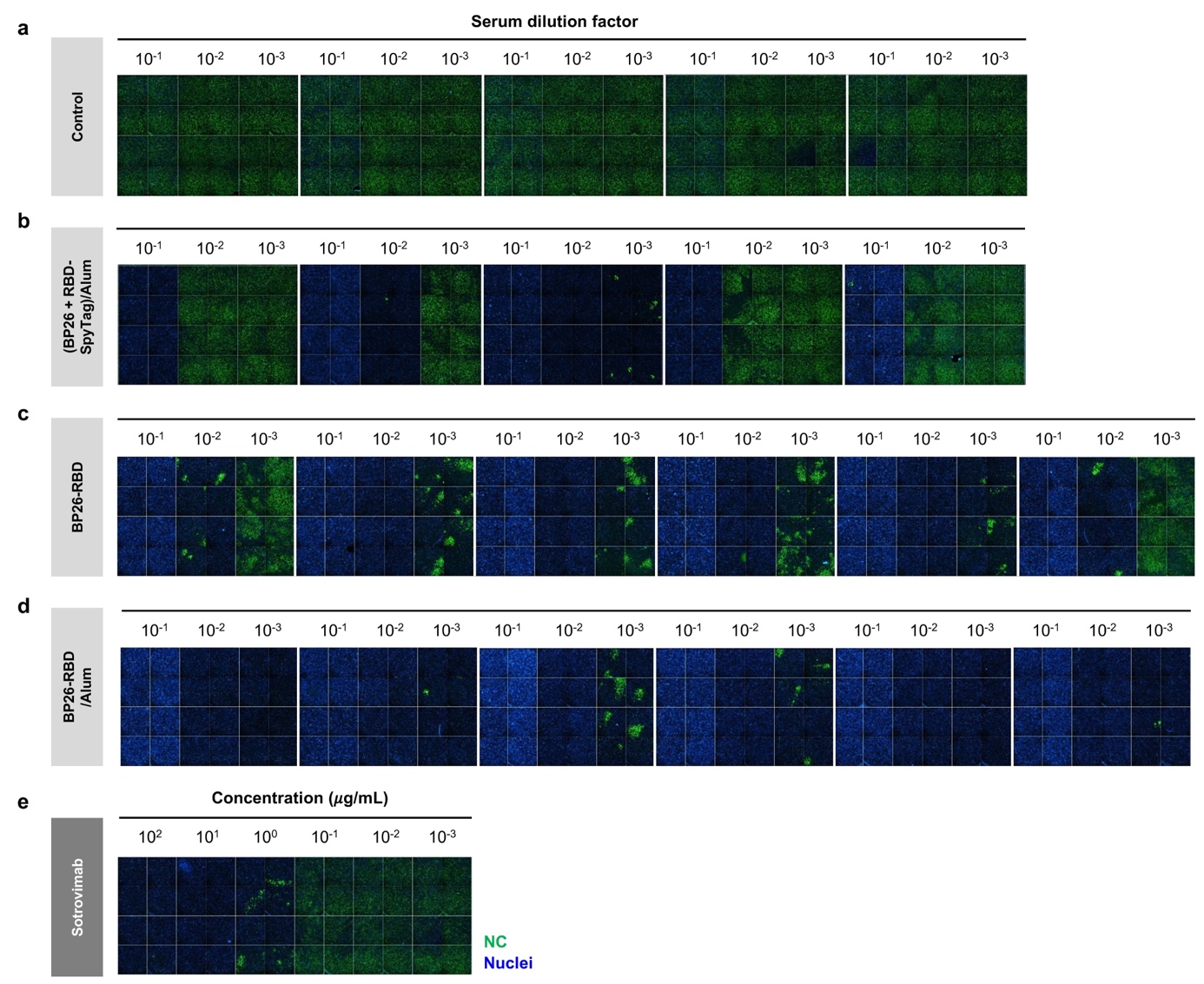


**Figure S6. Fluorescence imaging of Vero cells treated with preincubated mixtures of SARS-CoV-2 and sera from RBD-immunized mice.** In the absence of neutralizing antibodies, viral infection leads to nucleocapsid (NC) protein expression, detected by immunofluorescence using an anti-NC antibody and Alexa Fluor 488-conjugated secondary antibody (green). Cell nuclei were counterstained with DAPI (blue). Representative fluorescence images are shown for Vero cells treated with serum from mice immunized with: **a)** PBS (negative control), **b)** a physical mixture of BP26 nanoparticles and RBD-SpyTag with alum adjuvant, **c)** BP26-RBD conjugates without adjuvant, and **d)** BP26-RBD conjugates formulated with alum. **e)** Sotrovimab, a SARS-CoV-2–neutralizing monoclonal antibody, was used as a positive control. All neutralizing assays were performed in technical duplicates.


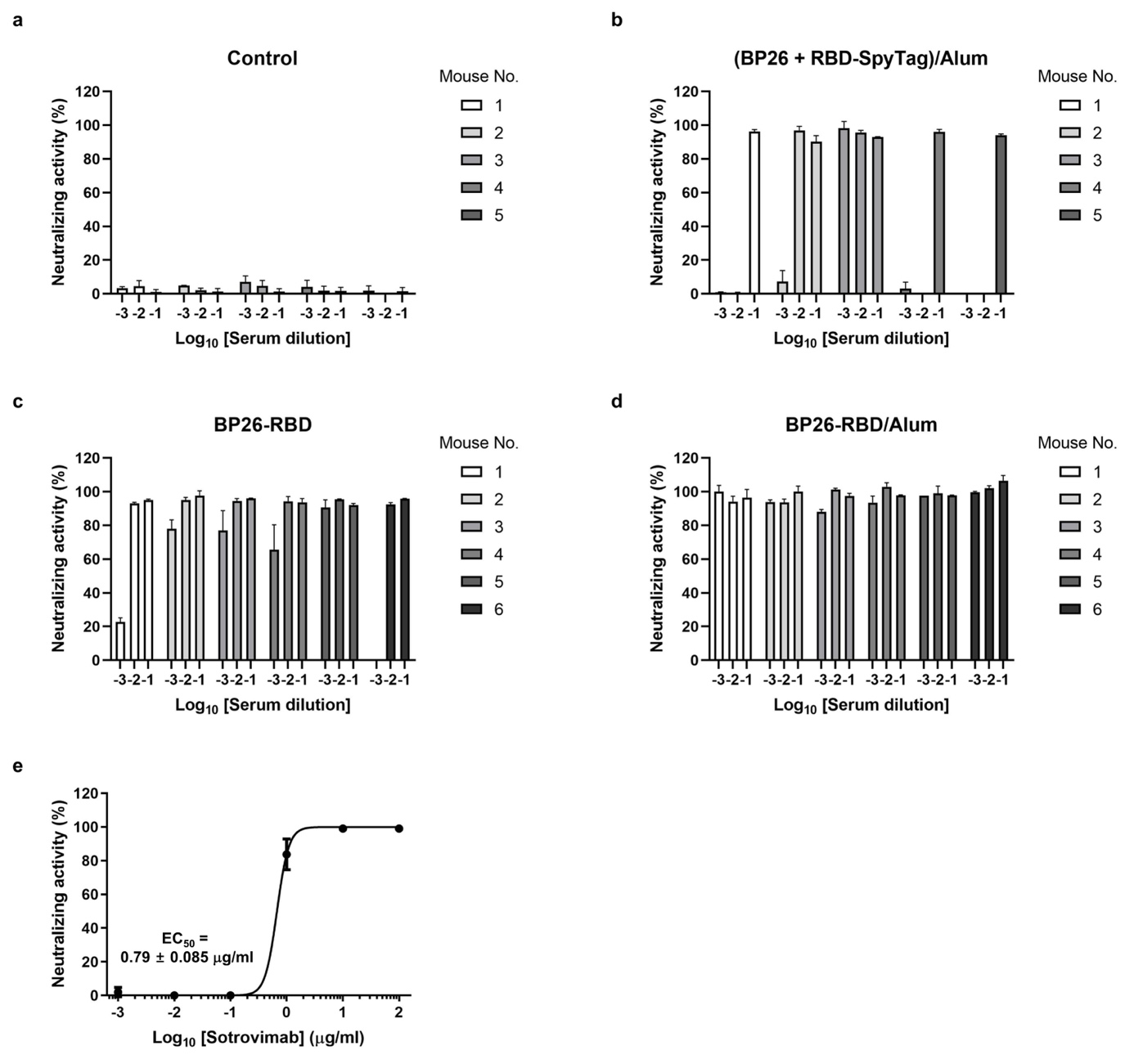


**Figure S7. Neutralizing activity of sera from individual immunized mice.** Neutralizing activity (%) was quantified by measuring the reduction in nucleocapsid (NC) protein expression in infected cells, normalized to the number of DAPI-stained nuclei. The following immunization groups were evaluated: **a)** PBS (negative control), **b)** physical mixture of BP26 nanoparticles and RBD-SpyTag with alum adjuvant, **c)** BP26-RBD conjugates without adjuvant, and **d)** BP26-RBD conjugates formulated with alum. **e)** Sotrovimab, a SARS-CoV-2–neutralizing monoclonal antibody, was included as a positive control. Data are presented as mean ± S.D. from technical duplicates.


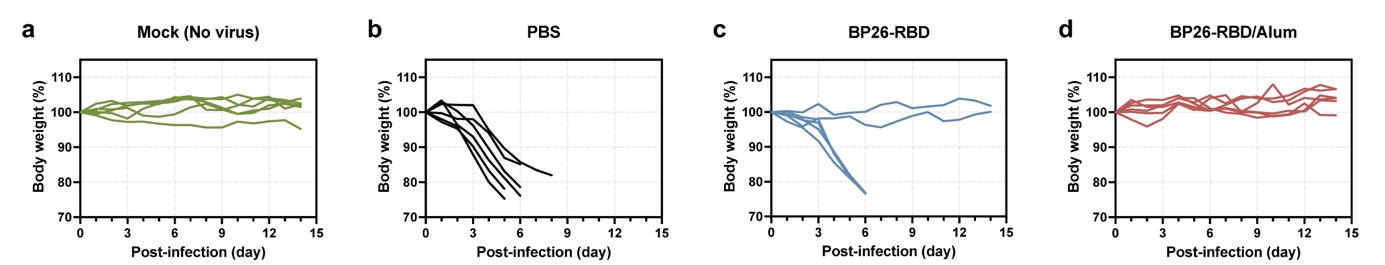


**Figure S8. Individual body weight trajectories following SARS-CoV-2 challenge.** K18-hACE2 transgenic mice (n = 6 per group) were immunized twice at a 3-week interval with various vaccine formulations. Two weeks after the booster immunization, mice were intranasally challenged with SARS-CoV-2 at a dose of 30 × median lethal dose 50% (MLD₅₀). Body weight was monitored daily for 14 days post-infection. Individual weight change curves are shown for: **a)** Unimmunized, unchallenged control mice (mock), **b)** SARS-CoV-2–challenged mice immunized with PBS (negative control), **c)** BP26-RBD without adjuvant, and **d)** BP26-RBD formulated with alum.


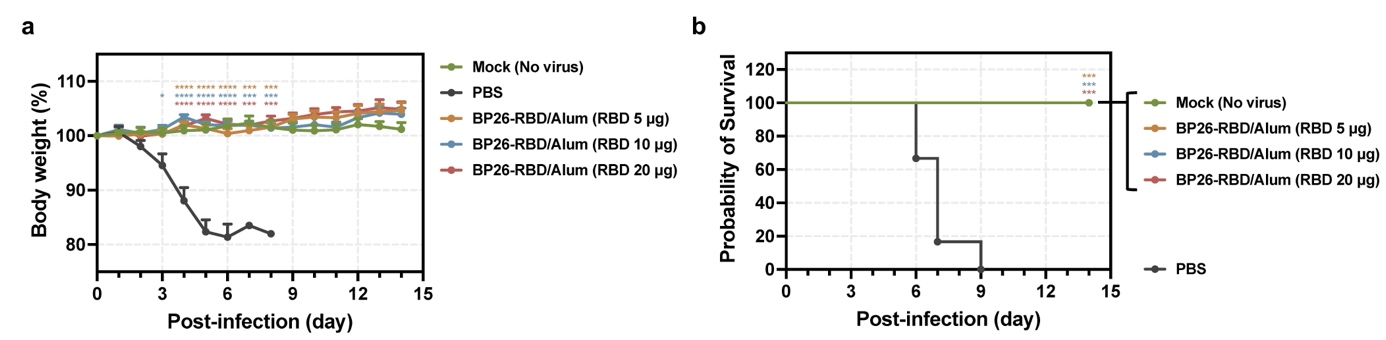


**Figure S9. Dose-dependent protective efficacy of BP26-RBD vaccination against SARS-CoV-2 challenge.** K18-hACE2 transgenic mice (n = 6 per group) were immunized twice at a 3-week interval with BP26-RBD formulated with alum adjuvant, containing 5 μg, 10 μg, or 20 μg of RBD per dose. Two weeks after the booster immunization, mice were intranasally challenged with SARS-CoV-2 at a dose of 30 × MLD₅₀. **a)** Body weight changes and **b)** survival rates were monitored over time post-challenge to evaluate dose-dependent protective efficacy. Data are presented as mean ± S.E.M. Statistical significance was determined versus the PBS group (**P* < 0.05, ****P* < 0.001, *****P* < 0.0001; multiple unpaired two-tailed t-tests with Holm-Sidak correction). Survival curves were analyzed using the Log-rank (Mantel-Cox) test.

**Table S1. Amino acid sequences and predicted molecular weights of BP26-SpyCatcher and RBD-SpyTag fusion proteins.** Shown are the full amino acid sequences and calculated molecular weights of the recombinant fusion proteins used for nanoparticle assembly: BP26-SpyCatcher and RBD-SpyTag. The sequences include all linker regions and fusion tags, as applicable.

| **Protein** | **Protein sequence** | **Molecular weight** |
| --- | --- | --- |
| **BP26**-**SpyCatcher** | GSQENQMTTQPARIAVTGEGMMTASPDMAILNLSVLRQAKTAREAMTANNEAMTKVLDAMKKAGIEDRDLQTGGIDIQPIYVYPDDKNNLKEPTITGYSVSTSLTVRVRELANVGKILDESVTLGVNQGGDLNLVNDNPSAVINEARKRAVANAIAKAKTLADAAGVGLGRVVEISELSRPPMPMPIARGQFRTMLAAAPDNSVPIAAGENSYNVSVNVVFEIKMVDTLSGLSSEQGQSGDMTIEEDSATHIKFSKRDEDGKELAGATMELRDSSGKTISTWISDGQVKDFYLYPGKYTFVETAAPDGYEVATAITFTVNEQGQVTVNGKATKGDAHI | **36.0 kDa** |
| **RBD**-**SpyTag** | RVQPTESIVRFPNITNLCPFGEVFNATRFASVYAWNRKRISNCVADYSVLYNSASFSTFKCYGVSPTKLNDLCFTNVYADSFVIRGDEVRQIAPGQTGKIADYNYKLPDDFTGCVIAWNSNNLDSKVGGNYNYLYRLFRKSNLKPFERDISTEIYQAGSTPCNGVEGFNCYFPLQSYGFQPTNGVGYQPYRVVVLSFELLHAPATVCGPKKSTNLVKNKCVNFGSGSGGAHIVMVDAYKPTKGS | **27.1 kDa** |

**Table S2. Molecular weight of the BP26 monomer and hydrodynamic size of BP26-based nanoparticles.** This table summarizes the theoretical molecular weight of the BP26 monomer and the hydrodynamic diameters of assembled BP26 and BP26-RBD, as measured by dynamic light scattering (DLS). Reported values include mean particle size and polydispersity index (PDI), illustrating the size increase following RBD antigen conjugation.

|  | **Molecular weight of monomer** [kDa] | **Hydrodynamic size** [nm] |
| --- | --- | --- |
| **BP26** | 23.6 | 11.0 |
| **BP26-M30** | 43.5 | 18.4 |
| **BP26-RBD** | 63.1 | 22.0 |
